# Novel Nicotinic Acetylcholine Receptor Inhibitors Derived from Oleoylcholine Analogs

**DOI:** 10.32607/actanaturae.27696

**Published:** 2026

**Authors:** M. V. Vladykina, I. S. Kokaeva, I. E. Kasheverov, N. M. Gretskaya, G. N. Zinchenko, V. I. Tsetlin, Yu. N. Utkin, V. V. Bezuglov, I. V. Shelukhina

**Affiliations:** Department of Molecular Neuroimmune Signaling, Shemyakin-Ovchinnikov Institute of Bioorganic Chemistry, Russian Academy of Sciences, Moscow, 117997 Russia

**Keywords:** acylated cholines, nicotinic acetylcholine receptor, inhibitor, DOTAP, SH-SY5Y, cytotoxicity

## Abstract

Fatty acid-acylated cholines, a recently identified class of endogenous compounds, have been de tected in both human and animal organisms. Our prior work established that oleoylcholine (Ol-Chol), and other acylcholines, at micromolar levels, modulate the cholinergic system and are suitable as cationic lipids for introducing nucleic acids into human and animal cells. The present research examines the interaction with the nicotinic acetylcholine receptors (nAChR) of two ionic forms of Ol-Chol and two synthesized cat ionic lipids, each featuring a quaternary ammonium moiety and two oleic acid chains. A radioligand bind ing assay revealed that the affinity of acylcholines and synthetic cationic lipids for the muscle-type nAChR surpasses that for the human neuronal α7 nAChR by a factor of 2–5.5. Oleoylcholine iodide demonstrated a two-fold higher efficacy of mesylate in binding to the orthosteric site of muscle and α7 nAChR. In a func tional calcium imaging assay, both compounds exhibited superior inhibition of α7 nAChR by several orders of magnitude, suggesting potential interaction with allosteric binding sites. Compared to oleoylcholine, syn thetic cationic lipids demonstrated markedly reduced efficacy in binding to α7 nAChRs and, in contrast to oleoylcholine, induced a substantial cytotoxic impact on SH-SY5Y neuroblastoma cells, a phenomenon unaf fected by specific nAChR ligands. As a result, the nAChR-inhibitory properties are attributed to the quater nary ammonium group present in all studied compounds. However, the modification of the lipophilic moiety with two oleic acid residues curbs these properties but enhances cytotoxic activity through an alternative mechanism independent of nAChR.

## INTRODUCTION

Acetylcholine analogs, defined as choline esters of un saturated fatty acids, have been detected in human plasma and urine [1]. Furthermore, elevated levels of long-chain unsaturated acylcholines are observed in specific pathological conditions. For instance, cho line esters of oleic, linoleic, and arachidonic acids have been detected within the vascular tissues of individu als with cardiovascular conditions, specifically abdom inal aortic aneurysms, atherosclerotic plaques in the carotid arteries, atherosclerotic plaques in the femoral arteries, and intimal thickening [2, 3]. Acylcholines, specifically their unsaturated forms, are present in elevated concentrations in the blood of individu als at a heightened risk for pulmonary embolism (PE) when contrasted with those at a moderate risk for the condition [4]. Accordingly, it is plausible that excessive accumulation of acylcholines is linked to the etiology of a range of human diseases. However, the precise mechanism by which acylcholines operate is not yet well-established.

Our prior research indicated that specific endog enous acylcholines, including arachidonoylcholine, oleoylcholine (Ol-Chol), linolenoylcholine, and docosa hexaenoylcholine, exhibit inhibitory effects on mus cle and α7 neuronal nicotinic acetylcholine receptors (nAChRs), as well as on acetylcholine-hydrolyzing en zymes, at micromolar concentrations [5]. This finding substantiates the hypothesis that the biological action of endogenous acylcholines is associated with their regulatory influence on acetylcholine-mediated sig naling pathways. Endogenous acylcholines may poten tially function as inhibitors of the oncogenic process in cancer cells that exhibit sensitivity to α7-nAChR inhibition, such as in lung cancer [6].

Due to their cationic lipid nature and the presence of a quaternary ammonium group, acylcholines can be used to deliver nucleic acids into mammalian cells [7]. DOTAP (1,2-bis-(9Z-octadecenoyl)oxy)-3-trimethyl ammoniumpropane) is a cationic lipid that has gained widespread popularity in the field of transfection re search due to its ability to facilitate the transfer of genetic material into cells, as evidenced by numer ous studies [8, 9]. This compound contains a charged quaternary ammonium group, a structural element that is analogous to choline esters. Despite the struc tural parallels between DOTAP and choline esters, the potential for this cationic lipid to engage with ace tylcholine receptors has not yet been investigated. Furthermore, our findings suggest that the efficiency of cellular transfection is dictated by the counterion present in the cationic lipid [7]. However, the impact of counterion properties on the inhibitory action of acylcholine on nAChRs remains to be elucidated.

**Fig. 1:**
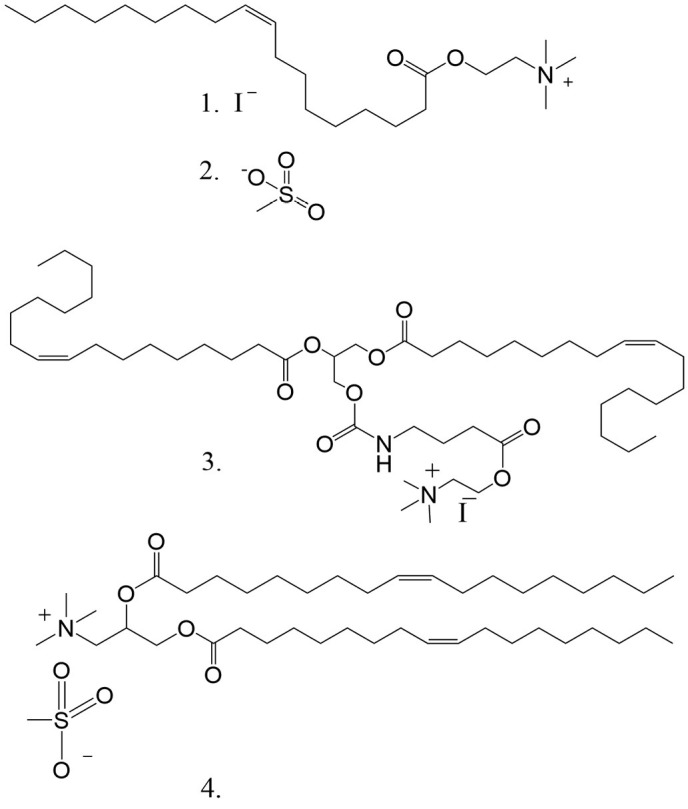
The structures of the acylcholines under study: (1) oleoylcholine iodide (Ol-Chol^+^I^-^), (2) oleoylcholine mesylate Ol-Chol^+^Mes^-^), (3) 4-((((1,3-bis(oleoyloxy)pro pan-2-yl)oxy)carbonyl)amino)butanoic acid choline ester iodide (DOGG-Chol^+^I^-^), (4) 1,2-Bis-(9Z-octadecenoyl) 3-trimethylammonium 1,2-dihydroxypropane mesylate (DOTAP^+^Mes^-^)

In this study, we investigated the interaction of two ionic forms of Ol-Chol and two synthetic cat ionic lipids containing a quaternary ammonium group and two oleic acid residues with muscle and neu ronal α7 nAChRs. To this end we synthesized two forms of oleoylcholine: iodide and mesylate. In addi tion, two other compounds were prepared: DOTAP mesylate and a new DOTAP analog containing a cho line group at a certain distance from the lipophil ic dioleoylglycerol residue: the choline ester iodide 4-((((1,3-bis(oleoyloxy)propan-2-yl)oxy)carbonyl)ami no)butanoic acid (Fig. 1). In contrast to oleoylcholine, both compounds contain two unsaturated fatty (oleic) acid residues.

nAChRs are ligand-gated membrane cation chan nels [10]. Muscle-type nAChRs are located on muscle f ibers at neuromuscular junctions and are responsible for transmitting nerve impulses to effector cells, while α7 nAChRs are widely distributed throughout the body and are present on both nerve cells and other cell types, such as immune cells, glial cells, epithelial cells, and others [11, 12, 13]. α7 nAChRs modulate a wide range of cellular processes, including the release of neurotransmitters, cytokines, and neurotrophic fac tors, as well as subsequent signal transduction, gene expression, and other processes [11, 14]. Dysfunction of muscle nicotinic acetylcholine receptors has been associated with the development of myasthenia gra vis. In contrast, α7 nicotinic acetylcholine receptors have been linked to a variety of conditions, including neurodegenerative and psychiatric disorders, chronic pain, sepsis, rheumatoid arthritis, and cancer [6, 12, 15]. Consequently, α7 nAChR is regarded as a molec ular target for drug development and targeted drug delivery [16, 17].

## EXPERIMENTAL

**Materials**

The following materials were used: dioleoyl glyceride (courtesy of E.L. Vodovozova, Shemyakin-Ovchinnikov Institute of Bioorganic Chemistry, Russian Academy of Sciences), N,N’-disuccinimidyl carbonate (DSC, Sigma-Aldrich, USA), gamma-aminobutyric acid (Acros Organics, Germany, > 99%), bis(trimethylsilyl) trifluoroacetamide (BSTFA, Acros Organics), Kieselgel 60 (Merck, USA), triethylamine, iodomethane (Acros Organics, > 99%), 1,1’-carbonyldiimidazole (1,1’-CDI, Fluka, Switzerland), dimethylaminopyridine (DMAP) (Fluka, Switzerland), dimethylaminoethanol (DMAE) (Sigma-Aldrich), sodium bisulfate (Fluka), anhydrous Na2 SO4 , ethyl acetate, benzene, dichloromethane, ace tonitrile, acetone, HCl, NaCl (Himmed, Russia).

The following reagents were used: the Fluo-4 Direct Calcium Assay Kit (ThermoFisher Scientific, USA), along with resazurin (Macklin Inc., Shanghai, China) and a variety of nAChR ligands, including nicotine (Sigma-Aldrich), mecamylamine, d-tubocu rarine, d-TC, methyllycaconitine, MLA, PNU120596, and PNU282987 (Tocris, UK). Additionally, the human neuroblastoma cell line SH-SY5Y (Sigma-Aldrich) was employed. The preparation of electric organ membranes from Torpedo californica was provided by F. Hucho (Free University of Berlin, Germany). The GH4C1 cell suspension was supplied by Eli Lilly, UK.

Synthesis of the iodide of the choline ester of 4-((((1,3-bis(oleoyloxy)propan-2-yl)oxy) carbonyl)amino)butanoic acid (DOGG-Chol^+^I^-^)

A solution was prepared by dissolving dioleoyl glycerol (100 mg, 0.16 mmol) in 1 mL of chloro form, followed by the addition of N,N’-DSC (45 mg, 0.18 mmol) and triethylamine (48 μL, 0.48 mmol). The mixture was stirred at 25°C for 20 minutes until the components were completely dissolved (Solution 1). Bis(trimethylsilyl)trifluoroacetamide (0.5 mL, 1.94 mmol) was added to a suspension of gamma-aminobutyric acid (50 mg, 0.5 mmol) in ace tonitrile (500 μL), followed by stirring at 25°C until complete dissolution of the gamma-aminobutyric acid. Subsequently, the resultant solution was incorporat ed into Solution 1. The reaction mixture was stirred for 18 h at +4°C. Extraction of the mixture was ac complished using ethyl acetate, followed by washing the extract with a 2 N sodium bisulfate solution, wa ter, and a saturated NaCl solution. The extract was then dried with anhydrous Na_2_SO_4_ , filtered, and con centrated through evaporation. The substance was obtained in the amount of 118 mg, presenting as a pale-yellow oil. The target product was purified via column chromatography utilizing a Kieselgel 60 sil ica gel, employing a benzene–ethyl acetate gradient from 0 to 15% ethyl acetate. An amount of 47 mg of 4-((((1,3-bis(oleoyloxy)propan-2-yl)oxy)carbonyl)ami no)butanoic acid (DOGG) was isolated in 39% yield as a colorless oil. ¹H NMR (CDCl3 ; m, J, 300 MHz) 0.90 (6H, s, 2H18), 1.28–1.32 (40H, m, 2H4; 2H5; 2H6; 2H7; 2H12; 2H13; 2H14; 2H15; 2H16; 2H17), 1.63 (4H, m, 2H3), 1.88 (2H, H3’ (GABA)), 2.03 (8H, m, 2H8; 2H11), 2.34 (4H, 2H2), 2.43 (2H, H2’ (GABA)), 3.27 (2H, m, H4’), 4.20–4.28 (4H, dm, 2H2’ (glycerol)), 4.89 (1H, H1’’ (glycerol)), 5.36 (4H, m, 2H9; 2H10).

The next reaction involved dissolving 36 mg (0.05 mmol) of DOGG in 500 μL of methylene chlo ride, followed by the addition of 10 mg (0.06 mmol) of 1,1’-CDI and 8 μL (0.06 mmol) of triethylamine. This mixture was then stirred at 22°C using a mag netic stirrer for a duration of 60 minutes. A total of 1.8 mg (0.015 mmol) of DMAP and 10 μL (0.1 mmol) of DMAE were added to the prepared imidazolide of 4-((((1,3-bis(oleoyloxy)propan-2-yl)oxy)carbonyl) amino)butanoic acid, and the resultant mixture was stirred for 18 h at 22°C under an argon atmosphere. Following dilution of the reaction mixture with chlo roform, it underwent sequential washes with 0.1 N HCl and H_2_O. The organic phase was then treated with a saturated NaCl solution and dried using an anhydrous Na2 SO4 . The organic layer underwent fil tration and subsequent evaporation. The product was purified by column chromatography (Kieselgel 60) with step-wise gradient elution using hexane–EA (9 : 1), hexane–EA (30 : 10), and chloroform–metha nol (10 : 1). A total of 19.7 mg, representing 48.6% of the target substance, was recovered as a colorless oil. The resulting product was dissolved in dry acetone for quaternization and treated with a 6-fold excess of iodomethane. The synthesis yielded 22.4 mg (96%) of DOGG-Chol^+^I^-^, which appeared as white crystals. ¹H NMR (CDCl3 ; m, J, 300 MHz) 0.90 (6H, s, 2H18), 1.28–1.32 (40H, m, 2H4–7; 2H12–17], 1.63 (4H, m, 2H3), 1.88 (2H, H3’ (GABA)), 2.03 (8H, m, 2H8; 2H11), 2.34 (4H, 2H2), 2.43 (2H, H2’ (GABA)), 3.27 (2H, m, H4’), 3.34 (2H, H1’ DMAE), 4.20–4.28 (6H, dm, 2H2’ (glyc erol), H2’ DMAE), 4.89 (1H, H1’’ (glycerol)), 5.36 (4H, m, 2H9; 2H10).

**Competitive radioligand binding assay**

Membranes derived from the electric organ of the Torpedo californica electric ray, which contain mus cle-type α1β1γδ nAChRs (with a terminal concen tration of toxin-binding sites at 0.52 nM), or cells from the GH4C1 cell line engineered to express hu man neuronal α7 nAChRs (with a final concentration of toxin-binding sites at 0.4 nM), underwent incuba tion with diverse concentrations of acylcholines. These were introduced as a solution in DMSO, maintained at room temperature within a binding buffer (com posed of 20 mM Tris-HCl, 1 mg/mL BSA, and adjust ed to pH 8.0) for 2 h 50 min or 3 h 30 min, respective ly. For each specific experimental data point, including the control conditions representing 100% and 0% bind ing, the final buffer was modified to match the peak DMSO concentration, ranging from 0.15 to 1.5% across different experimental sets, within the reaction me dium containing the acylcholines under examina tion. Subsequently, [^125^I]-αBgt was introduced in the samples at a final concentration of 0.2 nM, followed by an additional 5-min incubation period. Unbound [^125^I]-αBgt was eliminated from the reaction mixture via rapid filtration using GF/C filters (Whatman, UK), followed by three washes with the binding buffer (3 mL per wash). The filters used were pretreated with 0.25% polyethyleneimine. Binding was quanti f ied as 100% under conditions where the radioligand bound without any competitors. Nonspecific binding (0%) was quantified through a comparable experiment involving the incubation of muscle and α7 nAChR preparations with 9 μM α-cobratoxin (CTX) for a du ration of 2 h and 50 min or 3 h and 30 min, respec tively. The quantification of bound [^125^I]-αBgt was con ducted employing a Wallac Wizard 1470 γ-counter (GMI Inc., USA). The efficacy of the test compounds in interacting with their respective targets was quan tified through IC_50_ value analysis, utilizing OriginPro 2015 (Microcal, USA).

**Calcium imaging**

Human neuroblastoma SH-SY5Y cells were cultured in a growth medium consisting of a DMEM medium (“PanEco,” Russia), 10% fetal bovine serum (FBS, neoFroxx GmbH, Germany), penicillin (50 IU/mL), and streptomycin (50 μg/mL, PanEco) at 37°C in a CO_2_ in cubator (5% CO_2_ ). In preparation for calcium imaging, the cells were seeded into a black 96-well plate (SPL Life Sciences, South Korea) at a density of 5,000 10,000 cells per well, and cultivation proceeded un til an 80–90% confluent monolayer formed. Following this, the growth medium was substituted with a buff er solution containing 140 mM NaCl, 2 mM CaCl_2_ , 2.8 mM KCl, 4 mM MgCl_2_ , 20 mM HEPES, and 10 mM glucose, adjusted to pH 7.4. The cells underwent in cubation with the Fluo-4 Direct Calcium Assay Kit (ThermoFisher Scientific, USA) for 30 min in the dark at 37°C, and then for another 30 min at room tem perature. Prior to the introduction of the specific α7 nAChR agonist (200 nM PNU 282987 (Tocris, UK)), the SH-SY5Y cells were incubated for 20 min with solutions comprising the test compounds and a specif ic α7 nAChR positive modulator (10 μM PNU 120596 (Tocris)). Complete inhibition of the observed calcium responses was achieved by incubating the cells with a 1 μM solution of α-cobratoxin (CTX), a specific an tagonist of α7 nAChR, for 20 min.

A Hidex Sense Microplate Reader (Hidex, Finland) was employed to record changes in the fluores cence of the Fluo-4 dye (λex/em = 485/535 ± 10 nm). OriginPro 2017 (OriginLab Corporation, USA) was employed for the analysis of fluorescence intensity variations.

**Rezazurin test for assessing cellular metabolic activity**

SH-SY5Y cells were introduced into a transparent 96-well plate (SPL Life Sciences, Korea) at a seed ing density of 3,000 cells per well. Following a 24-h incubation period, the test compounds Ol-Chol+I-, Ol-Chol+Mes-, DOGG-Chol^+^I^-^, and DOTAP^+^Mes^-^ were introduced into the cellular cultures. These compounds were administered at concentrations ranging from 6.25 to 100 μM. Concurrently, nAChR ligands, specifi cally 100 μM nicotine (Nic, Sigma-Aldrich, Germany), mecamylamine (Mec), d-tubocurarine (d-TC), and methyllycaconitine (MLA); 10 μM PNU 120596, PNU 282987 (Tocris, UK); and 1 μM α-cobratoxin (CTX) were also added. The cells were cultured for 72 h, followed by a determination of cellular metabolic ac tivity using resazurin as previously described [18]. Following the removal of the culture medium, the cells were subjected to incubation for a duration of 4 h in the presence of a resazurin solution (Macklin Inc., China) prepared in a buffer formulated with 140 mM NaCl, 2 mM CaCl_2_ , 2.8 mM KCl, 4 mM MgCl_2_ , 20 mM HEPES, 10 mM glucose, and a pH of 7.4. Thereafter, the fluorescence intensity (λex/em = 550/590 ± 10 nm) of the resazurin reduction product (resorufin) was meas ured using a microplate fluorometer (Hidex, Finland). Wells without cells served as negative controls, while intact SH-SY5Y cells served as positive controls.

**Determination of extracellular lactate dehydrogenase (LDH) activity to assess the cytotoxicity of the compounds**

SH-SY5Y cells were seeded into a transparent 96-well plate (SPL Life Sciences, Korea) at a den sity of 3,000 cells per well. Following a 24-h incu bation period, the cells were subjected to treatment with Ol-Chol+I- and DOTAP^+^Mes^-^ at concentrations of 6.25–100 μM. After an additional 72 h of culture, extracellular lactate dehydrogenase activity was as sessed via the LDH Cytotoxicity Assay kit (Wuhan Servicebio Technology Co., Ltd., China), following the manufacturer’s protocol. Following a 30-min incuba tion period of an 80-μL aliquot of the selected cul ture medium with 80 μL of a working solution for lactate dehydrogenase detection, the optical density of the solution was assessed. This measurement was taken at λ = 490 nm using a Hidex Sense Microplate Reader (Hidex, Finland). Wells without cells served as negative controls, and lysed SH-SY5Y cells (Cell lysis buffer, LDH Cytotoxicity Assay kit) served as positive controls.

## RESULTS AND DISCUSSION

**Synthesis of acylcholines and their cationic dioleoyl analogs**

The compounds synthesized via chemical means and utilized in this study are shown in Fig. 1. The synthe sis of oleoylcholine in the form of iodide (Ol-Chol^+^I^-^) and mesylate Ol-Chol^+^Mes^-^) was conducted as de scribed previously [19]. DOTAP mesylate was syn thesized from 1,2-dimethylaminopropanediol-1,2 and oleic acid [20], followed by quaternization with dime thyl sulfate. DOGG-Chol^+^I^-^ was obtained from 1,2-di oleoylglycerol by the sequential introduction of GABA and DMAE into its structure followed by quaterniza tion with iodometane.

**Interaction of the synthesized compounds with nicotinic acetylcholine receptors**

The capacity of the synthesized compounds to inter act with the orthosteric binding site of nAChR was evaluated using a radioligand binding assay, and the functional activity of nAChR was determined by cal cium imaging.

**Fig. 2:**
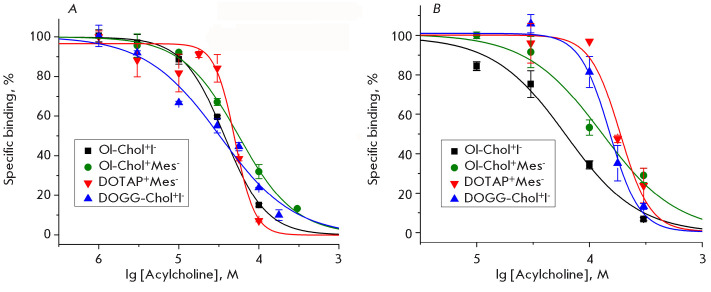
The dependence of the specific binding of radioactive [^125^I]-αBgt to the muscle nAChR of the ray Torpedo cali fornica (A) and human α7 nAChR (B) on the concentration of the acylcholines under study and their dioleoyl analogs. Each data point on the graphs represents the mean ± standard error of the mean, n = 2–3

To elucidate the interaction of iodide and me sylate oleoylcholine, alongside the cationic lipids DOGG-Chol^+^I^-^ and DOTAP^+^Mes^-^, with the orthostericbinding site of nAChR, we examined their competitive binding with [^125^I]-α-bungarotoxin ([^125^I]-αBgt) to the muscle nAChR from the electric organ of T. califor nica ray and to a human neuronal α7 nAChR via a radioligand binding assay (Fig. 2).

**Table 1:** The affinity of acylcholine analogs for two nAChR subtypes, presented as IC_50_ values (μM), calculated basing on a radioligand binding assay and Ca^2+^ imaging data

Acylcholines	nAChR T. californica	α7 nAChR	
Radioligand binding assay, IC_50_(μM)	Radioligand binding assay, IC_50_(μM)	Ca^2+^ imaging, IC_50_(μM)
Ol-Chol^+^I^-^	37 ± 1	62 ± 4	3.95 ± 0.18
Ol-Chol+Mes-	56 ± 2	129 ± 8	0.79 ± 2.24
DOGG-Chol^+^I^-^	50 ± 3	151 ± 4	7.32 ± 2.97
DOTAP^+^Mes^-^	32.5 ± 3.5	178 ± 8	14.99 ± 9.10

All the compounds were found to bind to the or thosteric sites of both receptors (Fig. 2). The affin ity of Ol-Chol^+^I^-^ and Ol-Chol^+^Mes^-^ and that of the dioleoyl analogs DOGG-Chol^+^I^-^ and DOTAP^+^Mes^-^ to the muscle-type receptors (IC_50_ range = 32.5–56 μM) was found to be 2.0–5.5 times higher than that to hu man α7 nAChRs (IC_50_ range = 62–178 μM) (Table 1). Concurrently, oleoylcholine iodide demonstrated a twofold efficacy in interacting with the orthosteric binding site of muscle and α7 nAChRs (IC_50_ = 37 and 62 μM, respectively) compared to the mesylate form (IC_50_ = 56 and 129 μM, respectively) (Table 1).

The binding affinity of the novel acylcholine ana logs Ol-Chol^+^Mes^-^, DOGG-Chol^+^I^-^, DOTAP^+^Mes^-^) for the muscle-type nicotinic acetylcholine receptor from the electric organ of T. californica was established via a radioligand binding assay, yielding IC_50_ values from 32.5 to 56 μM (Fig. 2 and Table 1). This affinity was found to be comparable to that of previously investi gated endogenous acylcholines (arachidonoylcholine, oleoylcholine, linolenoylcholine, and docosahexaenoyl choline), which exhibited IC_50_ values ranging from 18.7 to 93 μM [5]. It is of interest that the novel com pounds displayed lesser affinity (129–178 μM) for the human neuronal α7 subtype of nAChR compared to prior studies (14.2–80 μM) [5]. Our study is the first to demonstrate the effect of the nature of the counterion of a given acylcholine on the efficiency of its interaction with the receptor: oleoylcholine iodide ex hibited approximately a twofold affinity for its mesyl ate (Table 1).

**Fig. 3:**
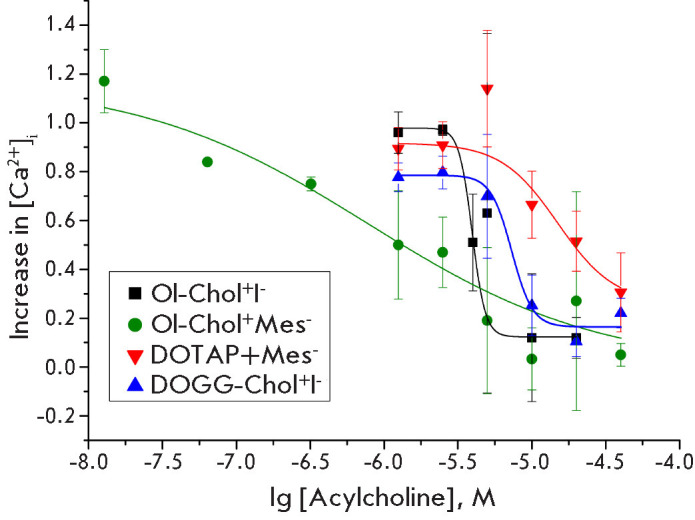
Inhibition by acylcholine analogs of the calcium responses induced by the activation of α7 nAChR with the specific agonist PNU282987 (200 nM) in SH-SY5Y neuro blastoma cells. The calculated inhibition parameters (IC_50_ ) are shown in Table 1. The data are presented as mean ± standard deviation, n = 4–6

Radioligand binding assays demonstrated that all the acetylcholine analogs under study bind to the α7 nAChR. Subsequently, calcium imaging was employed to examine their capacity to suppress the functional reactivity of this specific receptor subtype. For this purpose, SH-SY5Y neuroblastoma cells were used, which endogenously express α7 and several oth er nAChR subtypes [21]. All tested compounds were found to inhibit cellular responses induced by α7 nAChR activation with PNU282987, a specific agonist of this receptor, in the presence of the positive alloste ric modulator PNU120596 (Fig. 3). Oleoylcholine me sylate was identified as the most potent inhibitor, with an IC_50_ value of 0.79 μM. Its effectiveness dropped f ivefold when administered as iodide (Fig. 3, Table 1). DOGG-Chol^+^I^-^ and DOTAP^+^Mes^-^ were 9- and 19-fold less effective than Ol-Chol+Mes-, respectively (Fig. 3, Table 1).

This study provides the initial evidence of a sub stantial discrepancy in how the salt form of oleoyl choline modulates its inhibitory effect on nAChR. Oleoylcholine mesylate demonstrated the highest affinity for α7 nAChR and presented a significant ly flatter calcium response curve (Fig. 3, Table 1). Consequently, this compound demonstrated efficacy even at submicromolar concentrations (IC20 ~ 0.1 μM, IC_50_ = 0.8 μM). At the same time, the other com pounds investigated in the present study (Ol-Chol^+^I^-^, DOGG-Chol^+^I^-^, DOTAP^+^Mes^-^) and in our previously published work (iodides of arachidonoylcholine, linole noylcholine, and docosahexaenoylcholine) were active only in the micromolar concentration range (2–15 μM) (Table 1) [5].

Assessing the interaction of substances with the orthosteric ligand-binding site via a radioligand bind ing assay demonstrated that the affinity of all evaluated compounds for α7 nAChR was considerably lower than that determined by functional inhibition, as observed in calcium imaging (Table 1). This find ing suggests the potential involvement of alterna tive molecular mechanisms in the inhibitory action of acylcholine analogs, possibly through interaction with allosteric binding sites on the receptor. It is note worthy that increasing the complexity of the lipo philic moiety of oleoylcholine analogs (DOGG-Chol^+^I^-^ and DOTAP^+^Mes^-^) by introducing an additional oleic acid residue resulted in a decrease in the affinity of these compounds for the receptor. This reduction was evident in both radioligand binding assays (Fig. 2, Table 1) and calcium imaging experiments (Fig. 3, Table 1). The structural complexity of the receptor could potentially impede ligand interaction.

**The effect of oleoylcholine and its dioleoyl analogs on the viability and proliferative activity of SH-SY5Y neuroblastoma cells**

**Fig. 4:**
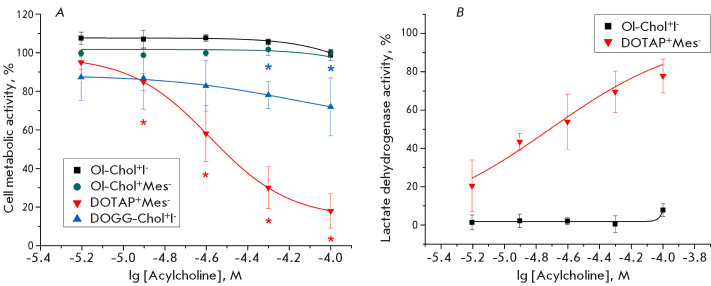
The effect of Ol-Chol^+^I^-^, Ol-Chol^+^Mes^-^, DOGG-Chol^+^I^-^ and DOTAP^+^Mes^-^ on the viability and proliferative activity of SH-SY5Y cells. The cells were incubated with various concentrations of the substances for 72 h. (A) the cellular met abolic activity was determined using the resazurin test; (B) the cytotoxicity of DOTAP^+^Mes^-^ and Ol-Chol^+^I^-^ was deter mined by quantifying extracellular LDH activity. The data are presented as mean ± standard deviation, n = 3. *p < 0.05 compared with control values, Mann–Whitney U-test

Several minutes are sufficient to assess the functional activity of oleoylcholine and its dioleoyl analogs with respect to α7 nAChR. However, the targeted intracellu lar delivery of nucleic acids and similar compounds re quires the incubation time of cationic lipids with cells to be extended to several hours or even days. In this regard, we conducted a comprehensive evaluation of the long-term effects (over 72 h) of oleoylcholine and its analogs DOGG-Chol^+^I^-^ and DOTAP^+^Mes^-^ on the vi ability and proliferation of SH-SY5Y neuroblastoma cells. This investigation included a thorough examina tion of the dependence of the observed effects on the activation or inhibition of nAChR using known ago nists, a positive allosteric modulator, and antagonists. For this purpose, the cellular metabolic activity was examined through the resazurin assay, and extracel lular lactate dehydrogenase activity was measured to assess the compound-induced cytotoxicity. Our find ings demonstrated that oleoylcholine (6.25–100 μM) did not elicit a significant alteration in cell viabil ity (as depicted in Fig. 4A,B). Conversely, the com pound DOGG-Chol^+^I^-^ demonstrated cytotoxic effects at all the concentrations tested (13–28% cell death, IC_50_ > 100 μM (Fig. 4A) ). The cytotoxic effect of the cationic lipid DOTAP^+^Mes^-^ exhibited a pronounced concentration-dependence (IC_50_ = 26.18 ± 0.16 μM in the resazurin assay (Fig. 4A) and 19.28 ± 5.64 μM in the LDH assay (Fig. 4B)).

Our prior findings [5] indicated that A549 lung ad enocarcinoma cells treated with oleoylcholine iodide (100 μM, 24 h) exhibited a concentration-dependent decrease in viability as a consequence of induced apoptosis, achieving a 45% rate. Although these cells demonstrate the presence of α7 nicotinic acetylcholine receptors [22], it is unlikely that this receptor subtype is responsible for the observed effect. This conclusion is supported by the finding that methyllycaconitine (10 μM), a specific inhibitor, did not alter the cytotoxic action of oleoylcholine [5].

**Fig. 5:**
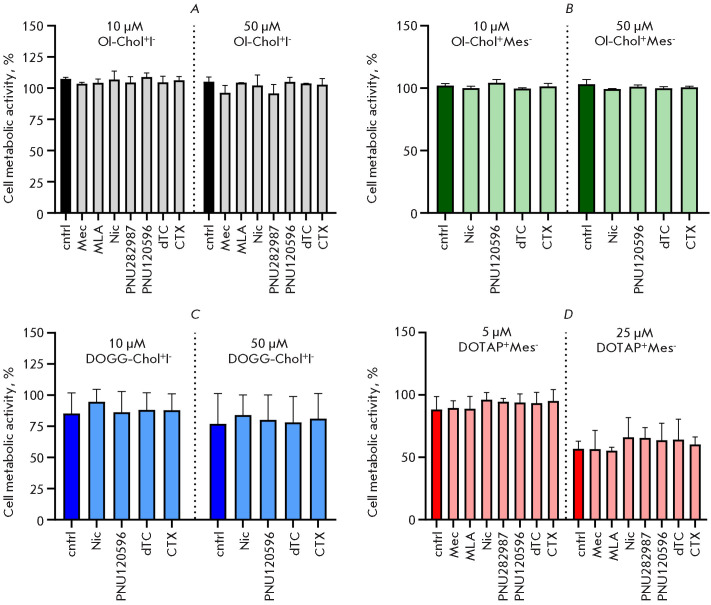
The effect of nAChR ligands on the long-term effects on the metabolic activity of SH-SY5Y cells over 72 h (A) Ol-Chol^+^I^-^, (B) Ol-Chol^+^Mes^-^, (C) DOGG-Chol^+^I^-^, and (D) DOTAP^+^Mes^-^. The nAChR ligands employed were: nicotine (Nic), mecamylamine (Mec), d-tubocurarine (d-TC), and methyllycaconitine (MLA) at a concentration of 100 μM; PNU120596 and PNU282987 at a concentration of 10 μM; and α-cobratoxin (CTX) at a concentration of 1 μM. The data are presented in relation to the metabolic activity values of intact SH-SY5Y cells. Cntrl is the relative metabolic activity of cells in the presence of the indicated concentration of (A) Ol-Chol^+^I^-^, (B) Ol-Chol+Mes-, (C) DOGG-Chol+I,- and (D) DOTAP^+^Mes^-^. The data are presented as the mean ± standard deviation, n = 3. *p < 0.05, Mann–Whitney U test, compared with control values

This investigation sought to determine if the en during impact of the examined cationic lipids on SH-SY5Y neuroblastoma cell viability is attributable to their interaction with nAChRs. With this objective in mind, SH-SY5Y cells were subjected to incubation for 72 h with the lipids under investigation at two distinct concentrations below their IC_50_ for cytotoxic activity (10 and 50 μM for Ol-Chol^+^I^-^, Ol-Chol+Mes-, DOGG-Chol^+^I^-^; 5 and 25 μM for DOTAP^+^Mes^-^), ac companied by the introduction of specific nAChR li gands (Fig. 5). SH-SY5Y cells have been observed to express homopentameric α7 nAChR, as well as vari ous heteropentameric nAChRs formed by combina tions of α3-, α5-, β2-, and β4-subunits [21]. The experimental findings demonstrate that non-selective agonists (nicotine), antagonists (mecamylamine and d tubocurarine), and specific α7 nAChR ligands (e.g., the agonist PNU282987, the positive allosteric modulator PNU120596, and antagonists such as methyllycaconi tine and α-cobratoxin) did not alter the effects of the examined compounds on SH-SY5Y cells (Fig. 5).

## CONCLUSION

Acylated cholines represent a recently discovered class of endogenous fatty acid analogs of acetyl choline which remain poorly studied. Our previ ous research demonstrated that choline esters of unsaturated fatty acids function as modulators of the acetylcholine system. The present work utilizes oleoylcholine, which earlier demonstrated the most significant activity in inhibiting α7 nAChR, to pro vide the first evidence that the counterion of the quaternary ammonium group substantially impacts the compound’s capacity to interact with nAChR. According to radioligand analysis of [^125^I]-αBgt bind ing to the orthosteric binding site of nAChR, oleoyl choline iodide interacted with muscle and neuronal α7 nAChR twice as effectively as mesylate. These results suggest that, under biological experimental conditions in the working buffer solution, the coun terion is not replaced by chloride despite its excess. Furthermore, the size of the counterion appears to significantly influence the interaction of acylcho line with the orthosteric acetylcholine binding site on the receptor. Utilizing calcium imaging, a meth od that captures the functional response of cells to nAChR activation, it was demonstrated that both salt forms of oleoylcholine (iodide and mesylate) were significantly more potent inhibitors of α7 nAChR (IC_50_ = 3.95 and 0.79 μM, respectively) than was ob served in experiments involving competition with [^125^I]-αBgt. In this case, the enhanced inhibitory ac tivity of oleoylcholine derivatives can be postulat ed to be associated with their additional interaction with allosteric binding sites on the receptor mole cule, which is not detected by a radioligand-based analysis.

A significant finding of this study is the discov ery that DOTAP, a cationic lipid widely used in lip id nanoparticles for the delivery of nucleic acids in side mammalian cells, possesses the capacity to inhibit both muscle-type and neuronal-type nAChRs. The potential of DOTAP, a cationic lipid, to modify acetyl choline receptor activity, though less pronounced than that of oleoylcholine, should be considered in the de sign of medications incorporating this compound. In contrast to oleoylcholine, the lipophilic component of DOTAP features two oleic acid residues. In the pres ent study, an oleoylcholine analog was synthesized in which the lipophilic moiety also contained two oleic acid residues within the 1,2-diacylglycerol backbone. The choline group was separated from the lipophil ic moiety by a GABA-based linker (DOGG-Chol^+^I^-^). Similar to DOTAP, this analog demonstrated affin ity for α7 nAChR, as substantiated by radioligand binding assays and functional responses in calcium imaging experiments. Nevertheless, its activity was considerably less pronounced than that of oleoylcho line. Whereas oleoylcholine fosters the proliferation of SH-SY5Y neuroblastoma cells, DOGG-Chol^+^I^-^ and DOTAP^+^Mes^-^ induced a significant cytotoxic effect on these cells. This effect, however, was not mediated by interaction with nAChRs, since specific nAChR li gands were unable to block it.

In summary, the choline group common to all the acylcholines under study makes them act as nAChR inhibitors. Conversely, the modification of the lipo philic domain of the molecule with an additional oleic acid group has been shown to attenuate its inhibitory function while amplifying its cytotoxic action, a pro cess that occurs through a mechanism independent of nAChRs.

## Abbreviations

nAChR: nicotinic acetylcholine receptor
αBgt: α-bungarotoxin from the venom of Bungarus multicinctus
[125I]-αBgt: α-bungarotoxin, labeled with the iodine-125 isotope
CTX: α-co bratoxin from Naja kaouthia venom
LDH: lactate dehydrogenase
Ol-Chol+I-: oleoylcholine iodide
Ol-Chol+Mes-: oleoylcholine mesylate
DOTAP+Mes-: (1,2-Bis-(9Z-octadecenoyloxy)-3-trimethylammonium propane mesylate
DOGG-Chol+I-: choline ester iodide of 4-((((1,3-bis(oleoyloxy)propan-2-yl)oxy)carbonyl) amino)butanoic acid
Nic: nicotine
Mec: mecamylamine
dTC: d-tubocurarine
MLA: methyllycaconitine
GABA: gamma-aminobutyric acid
